# Endometriosis and risk of depression among oral contraceptive users: a pooled analysis of cohort studies from 13 countries

**DOI:** 10.1093/humrep/deae299

**Published:** 2025-01-12

**Authors:** P De Corte, I Milhoranca, A S Oberg, T Kurth, S Mechsner, K Heinemann

**Affiliations:** Berlin Center for Epidemiology and Health Research, Berlin, Germany; Institute of Public Health, Charité—Universitätsmedizin Berlin, Berlin, Germany; Berlin Center for Epidemiology and Health Research, Berlin, Germany; Department of Medical Epidemiology and Biostatistics, Karolinska Institutet, Stockholm, Sweden; Institute of Public Health, Charité—Universitätsmedizin Berlin, Berlin, Germany; Endometriosis Centre Charité, Department of Gynaecology, Charité—Universitätsmedizin Berlin, Berlin, Germany; Berlin Center for Epidemiology and Health Research, Berlin, Germany

**Keywords:** endometriosis, epidemiology, contraception, psychology, dysmenorrhea, endometrium

## Abstract

**STUDY QUESTION:**

Does endometriosis affect the mental health of women using oral contraceptives?

**SUMMARY ANSWER:**

Among oral contraceptive users, women with endometriosis have a higher risk of depression compared to those without endometriosis, although the absolute risk increase is small.

**WHAT IS KNOWN ALREADY:**

Previous studies have suggested a potential link between endometriosis and mental health issues, but the impact of endometriosis on depression among oral contraceptive users remains unclear.

**STUDY DESIGN, SIZE, DURATION:**

A secondary pooled cohort study utilizing data from two longitudinal patient-centric studies (INAS-VIPOS and PRO-E2) was conducted across 11 European countries, Colombia and Australia. The study included 93 541 women newly prescribed oral contraceptives, with or without endometriosis, and without a self-reported history of depression.

**PARTICIPANTS/MATERIALS, SETTING, METHODS:**

Participant’s mental health was captured using self-administered questionnaires at baseline and every 6–12 months thereafter, asking about any newly occurred episodes of depression. Incidence rates (IRs) of self-reported depression were calculated per 10 000 woman-years. Absolute risk difference (ARD) and number needed to harm (NNH) were calculated with 95% CIs. The association between endometriosis and self-reported depression was estimated through crude and adjusted hazard ratios (HRs) with 95% CI, using stabilized inverse probability of treatment weighting (IPTW).

**MAIN RESULTS AND THE ROLE OF CHANCE:**

Of the included 93 541 women, 21 090 had endometriosis (49 541 woman-years) and 72 451 had no endometriosis (137 137 woman-years.) Of those with endometriosis, 308 (1.5%) reported an episode of depression (IR: 62.2/10 000, 95% CI: 55.4–69.5) compared to 535 (0.7%) of women without endometriosis (IR 39.0/10 000, 95% CI: 35.8–42.5). The ARD and NNH were 23.2 per 10 000 (95% CI: 15.2–30.9) and 431 (95% CI: 323.7–657.0) respectively. The HR of depression in women with endometriosis was 1.85 (95% CI: 1.60–2.13) using stabilized IPTW to control for age, BMI, smoking, education, and age at menarche. Subgroup and sensitivity analyses showed similar results.

**LIMITATIONS, REASONS FOR CAUTION:**

While efforts were made to control for confounding factors, residual confounding may still exist. Additionally, the results can only be generalized to users of oral contraceptives.

**WIDER IMPLICATIONS OF THE FINDINGS:**

These results highlight the importance of considering the mental health implications of endometriosis among women using oral contraceptives. Further research is needed to explore additional contributing factors and potential interventions.

**STUDY FUNDING/COMPETING INTEREST(S):**

No funding was received for this study. No competing interests apply for this research.

**TRIAL REGISTRATION NUMBER:**

N/A.

## Introduction

Endometriosis, a heterogeneous chronic condition characterized by the growth of endometrial-like tissue outside the uterine cavity (Horne *et al.*, 2017), is often considered a benign gynecological condition. However, endometriosis significantly impacts the health of women. While endometriosis is estimated to affect about 10% of individuals of reproductive age (Eskenazi and Warner, 1997), recent studies suggest this may be an underestimate given the lack of data on under-researched populations (Koninckx *et al.*, 2021; Moradi *et al.*, 2021).

The most commonly reported symptoms include pelvic pain, painful menstruation, and infertility, along with symptoms that are not directly linked with endometriosis such as gastrointestinal problems, fatigue, back pain, and painful urination. These symptoms often align with the menstrual cycle, leading to recurring flares of painful manifestations that could result in poor emotional health when not managed properly in clinical practice (Jones *et al.*, 2004; Nnoaham *et al.*, 2011; Moradi *et al.*, 2014; Parasar *et al.*, 2017).

Oral contraceptives (OCs) are commonly prescribed to relieve endometriosis-related pain symptoms, thus aiming to improve patient’s quality of life. Previous research found an association between endometriosis and major depression, especially those having persistent chronic pain (Chen *et al.*, 2016; Laganà *et al.*, 2017). A recent systematic review showed a significant relationship between endometriosis and depression, but this relationship was not evident when comparing only those with pelvic pain (PP) (among those with endometriosis) and those with no endometriosis (Gambadauro *et al.*, 2019).

Research on the relationship between endometriosis and mental health under OC treatment is limited. We hypothesize that OC users with endometriosis have a higher risk of developing depression compared to women without endometriosis. Therefore, the aim of this study was to understand the relationship between endometriosis and patient’s perceptions of depression in new users of OCs. This was investigated by comparing incidence rates (IRs) of self-reported depression between initiators of a new OC for those with and without endometriosis. In addition, the aim was to investigate if there is a causal relationship between endometriosis and self-reported depression, and if this is mediated by PP or painful menstruation. Since depression is hypothesized to be influenced by factors beyond pain, including genetic predisposition, socio-economic status, lifestyle, and psychosocial stressors, we further aimed to describe potential reasons, other than pain, affecting mental health.

## Materials and methods

This study was designed as a pooled cohort study, combining data from two international prospective post-authorization safety studies; INAS-VIPOS (Heinemann *et al.*, 2020) and PRO-E2 (Reed *et al.*, 2021) conducted by ZEG Berlin and comprising baseline and follow-up data of 27 840 and 124 920 female study participants respectively. Identical methodology, data collection tools, type of information collected, data management, and (medical) follow-up procedures allowed the data from the two studies to be pooled. The start of study observation in INAS-VIPOS was when women of any age diagnosed with endometriosis were prescribed a new treatment by their healthcare practitioner such as OCs, including progestogen-only pills, danazol, and gonadotropin-releasing hormone analogs. For PRO-E2, the start of observation was when women of any age were prescribed a new OC treatment for any reason (i.e. contraceptive reasons, endometriosis, acne, menstrual problems). The only exclusion criteria included unwillingness to sign consent and being unable to understand the study documentation. Both studies followed a patient-centric methodology, with self-administered baseline and follow-up questionnaires submitted every 6–12 months. Information obtained at baseline included demographics (age, weight, height, education), medical and gynecological history, current mood, presence of any serious disease, prior depression, current medication, and lifestyle (i.e. smoking). At each follow-up, women were asked about changes in treatment prescribed at baseline, regular use of concomitant medication, current mood, any hospitalization, any new serious disease (including depression), and pregnancy and/or delivery. This study includes data from participants coming from 13 countries (Austria, Australia, Colombia, France, Germany, Hungary, Italy, Poland, Russia, Spain, Sweden, Switzerland, and Ukraine). Further details about the data sources can be found in Supplementary Table S1 and are described elsewhere (Heinemann *et al.*, 2020; Reed *et al.*, 2021).

### Eligibility for the pooled study

**Figure 1.**
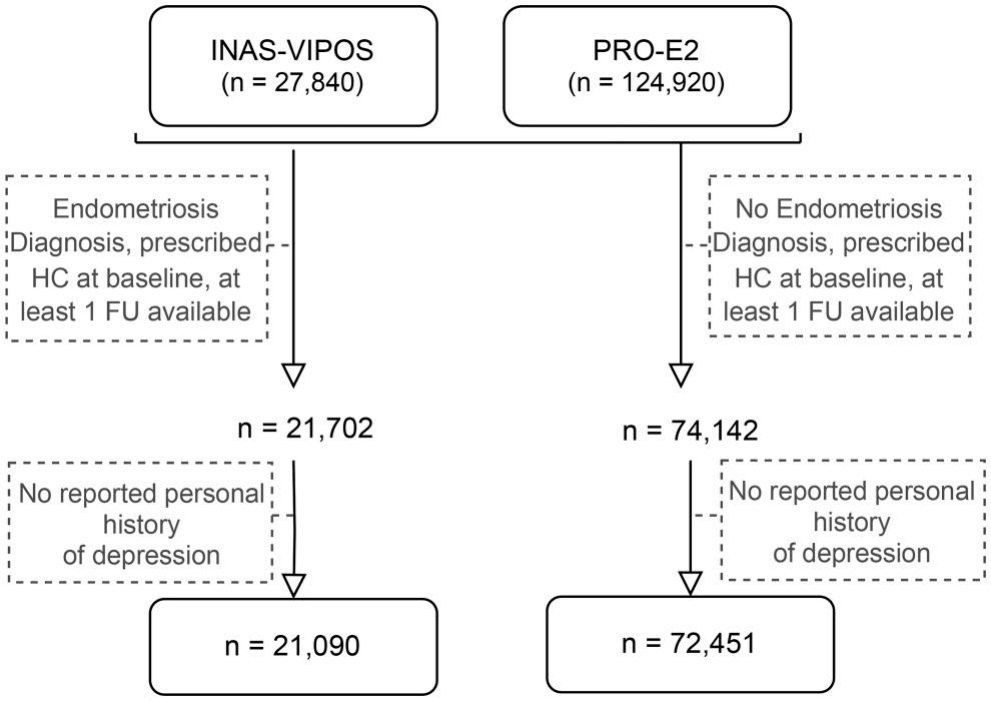
**Study participation flowchart.** HC, hormonal contraceptive (prescribed oral contraceptive); FU, follow-up.

Since the primary research interest concerned development of a newly reported episode of depression rather than potential worsening or reoccurrence of prior depression, study participants with a personal history of depression were excluded from this study. Additionally, given the potential difference in occurrence of undesired effects on mental health of some medications indicated for treating endometriosis, women initiating another treatment than OCs at baseline (i.e. danazol or gonadotropin-releasing hormone analogs) were not considered for the analyses. More details on study selection can be found in Fig. 1.

### Assessment of endometriosis

Women were identified as having endometriosis if the baseline participant of physician data in INAS-VIPOS and PRO-E2 indicated that (i) the woman had been diagnosed with endometriosis, or (ii) the woman was prescribed a new hormonal treatment including OCs for endometriosis, or (iii) the woman reported having been treated for endometriosis by a healthcare professional. If a participant did not meet any of the above criteria, this participant was classified in the non-endometriosis group.

### Assessment of depression

If women answered yes to the question ‘Have you been diagnosed with a depression requiring treatment’ during follow-up, the ZEG Berlin medical team obtained further medical documentation. In both studies, depression was considered as ‘confirmed’ if a woman was diagnosed by a psychiatrist, was admitted to the hospital due to their depression, or had attempted suicide. Other self-reports were considered as ‘not confirmed’ (e.g. depression treated by a psychologist, neurologist or general practitioner, depression during pregnancy, potential depression, other mental health conditions, and misunderstandings). At the end of each study, an independent expert committee reviewed the self-reports of depression and made a final categorization until consensus was reached. For this study, the primary interest concerned all self-reports of depression categorized as ‘confirmed’ and ‘not confirmed’, excluding not confirmed events categorized as misunderstandings. These include questions that were misunderstood by participants and erroneously marked ‘Yes’ on the questionnaire.

### Assessment of pain

Study participants with pelvic or menstrual pain were identified from baseline reporting that they suffer from ‘pelvic pain’, ‘painful periods’, or ‘painful menstrual bleeding’ prior to their start of OC treatment.

### Details of ethics approval

The planning and execution of the studies adhered to the national laws and regulations applicable in the participating countries. Prior to data documentation, informed consent was obtained from all study participants. The informed consent forms complied with the specific laws and regulations governing observational studies in each respective country, including obtaining approval from the local IEC/IRB. All studies were conducted in accordance with established guidelines, such as the Guidelines for Good Pharmacoepidemiology Practices from the International Society for Pharmacoepidemiology, the Good Epidemiological Practice from the International Epidemiological Association European Federation, and the ethical principles based on the Declaration of Helsinki. Furthermore, the studies adhered to the European Network of Centres for Pharmacoepidemiology and Pharmacovigilance (ENCePP) Code of Conduct for Scientific Independence and Transparency. The PRO-E2 (EUPAS 2196) and INAS-VIPOS (EUPAS 1613) studies were registered in the EU PAS and received the ENCePP seal. Additionally, both studies were registered on clinicaltrials.gov to ensure transparency and compliance.

### Statistical analysis

For the description of baseline and follow-up characteristics, categorical variables were presented by absolute and relative frequencies while continuous variables were summarized by the sample mean and standard deviation (SD).

IRs were calculated per 10 000 woman-years. Women contributed time at risk until the first self-reported depressive event, loss to follow-up or death, or when they reached 35 months of study follow-up, whichever occurred first. Additionally, we presented mean absolute risk differences (ARDs) and the number needed to harm (NNH) with 95% CIs over the complete follow-up time to increase informed decision-making for both patients and physicians (Bretthauer and Kalager, 2023). Bootstrap methods were employed to calculate the 95% CIs of the absolute risk estimates. Specifically, 500 bootstrap samples were generated using resampling with replacement, with each sample containing the same number of observations as the original dataset (Efron and Tibshirani, 1994).

To estimate the average effect of endometriosis on depression, inverse probability of treatment weighting (IPTW) was used. IPTW is a method that accounts for measured confounding in which, for each participant, a weight is calculated by inverting the estimated probability of their actual exposure status (i.e. endometriosis or not) given their confounder profile (Austin, 2014). The calculated weights determine to which extent participants contribute to a so-called pseudo-population, in which the distribution of observed measurable differences is balanced, thus mimicking a randomized trial. To increase statistical efficiency, stabilized weights were used (Robins *et al.*, 2000; Cole and Hernán, 2008).

To estimate the stabilized weights, we fitted a logistic model with exposure as the outcome, including the confounding variables. We then calculated stabilized weights for each individual. This was done by determining the ratio of the predicted probability of exposure (or one minus the probability of exposure if the individual was not exposed) from the intercept-only logistic regression model to the predicted probability of exposure (or one minus the probability of exposure) from the logistic regression model that included the confounding variables as predictor variables.

Confounding variables were identified using a causal diagram (Greenland *et al.*, 1999), which was developed in conjunction with currently available subject-matter knowledge and expert reviews (Supplementary Fig. S1).

Standardized mean differences (SMDs) were used to assess balance of the baseline variables between those with endometriosis and those without, before and after applying the weighting (Austin and Stuart, 2015). Adequate balance was considered when a variable’s absolute SMD was lower than 0.25 and its variance ratio fell between 0.5 and 2.0 (Rubin, 2001; Austin, 2017). In cases of residual imbalance, the logistic model was modified by introducing interactions or incorporating non-linear terms of the selected covariates. Once adequate balance between the groups was confirmed, weighted Cox proportional hazards regression was used to estimate IPT-weighted hazard ratios (HRs) with 95% CI to express relative risks. The Efron technique was employed to optimize ties in the analysis. Missing data on covariates were handled with multiple imputation using the fully conditional specification method (20). Ten imputed datasets were created (PROC MI, SAS 9.4). The results from the Cox regression analysis across the imputed datasets were combined using Rubin’s rules (PROC MIANALYZE, SAS 9.4) (Cole and Hernán, 2004). To assess diagnostics of the stabilized weights across the imputed datasets, the mean and range of the weights were summarized. Furthermore, IPTW-adjusted survival curves using the imputed dataset were generated to illustrate the depression probabilities for each treatment group over time (Cole and Hernán, 2004).

To investigate the potential impact of pelvic or menstrual pain, a subgroup analysis was done in women who had not reported these conditions at baseline. Sensitivity analyses explored the potential influence of misclassification, first by limiting the outcome to confirmed depression only, and second by limiting the exposure to surgically confirmed endometriosis. To assess the impact of selection bias, analyses were repeated while stratifying by new users and previous users of OC.

Lastly, factors that were assessed during baseline (endometriosis only) and follow-up (both exposure groups) that could contribute to worse mental health were summarized. SAS 9.4 was used for all data preparation and analyses.

## Results

**Figure 2.**
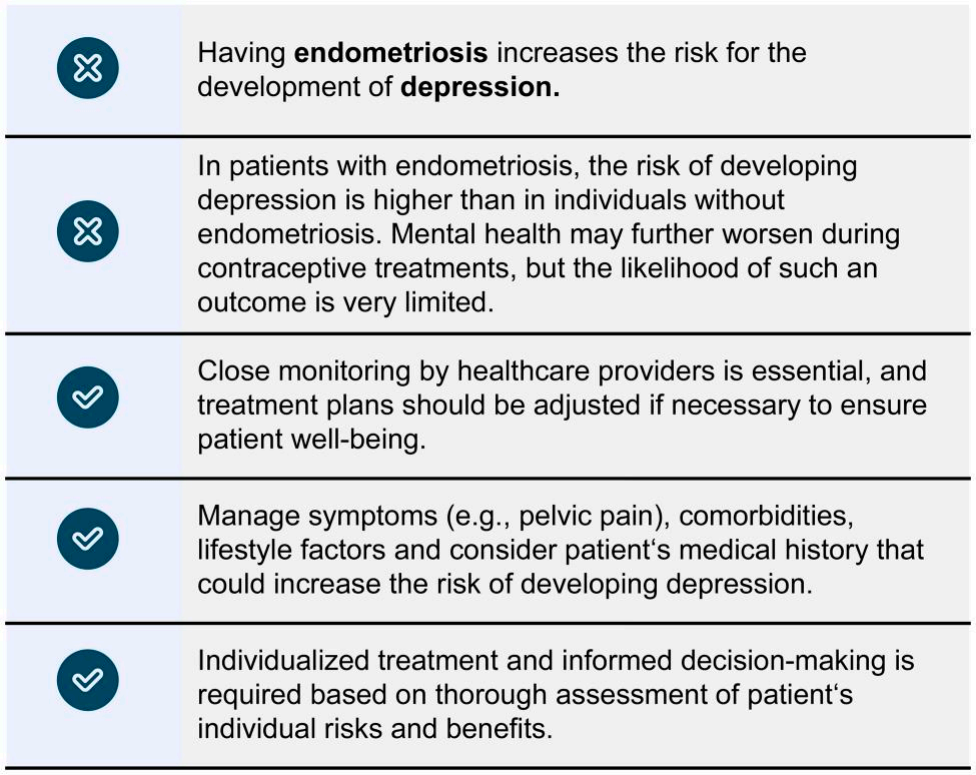
Summary of key findings on endometriosis and depression.

A visual summary of key findings in this research can be found in Fig. 2.

From the pooled dataset, 21 090 women with endometriosis and 72 451 women without endometriosis met the eligibility criteria, resulting in an available observation time of 49 541 and 137 137 woman-years respectively.

Overall, women with endometriosis had a higher mean age compared to unaffected women (32.8 vs 29.9 years) and were more likely to have an education higher than university entrance level (45.9% vs 39.9%). They were also more likely to be OC-naïve (24.4% vs 40.2% had used an OC before), report a history of a medical condition or disease (11.5% vs 5.5%), and use medication regularly (11.5% vs 8.1%). Other measured baseline characteristics were similar (Table 1).

**Table 1.** Baseline characteristics of the study population according to endometriosis status.

Number of participants	Endometriosis		No endometriosis	
	21 090		72 451	
**Patient characteristics**				
Age at study entry (years)	32.8	(±8.92)	29.9	(±8.56)
Weight at study entry (kg)	64.4	(±12.04)	63.0	(±11.94)
Height at study entry (cm)	166.1	(±5.92)	164.8	(±6.27)
BMI at study entry	23.4	(±4.24)	23.2	(±4.18)
**Gynecological history**				
Age at menarche (years)	12.9	(±1.32)	12.8	(±1.46)
Ever been pregnant (gravidity)	11 799	(56.0%)	39 907	(55.1%)
Ever given live birth (parity)	10 893	(51.7%)	36 099	(49.8%)
Number of live births	1.51	(±0.66)	1.60	(±0.74)
Number of miscarriages, abortion, or stillbirth	2.02	(±1.38)	1.58	(±1.01)
Ever used oral contraceptive	5145	(24.4%)	29 147	(40.2%)
**Lifestyle**				
Smoking	4118	(19.5%)	15 243	(21.0%)
Heavy smoking (>15 cigarettes per day)	504	(2.4%)	2245	(3.1%)
**Medical history**				
Reported disease or condition	2426	(11.5%)	3979	(5.5%)
**Medication**				
Regular use of medication	2425	(11.5%)	5879	(8.1%)
**Education**				
Higher than university entrance level	9678	(45.9%)	28 925	(39.9%)

### Depression absolute numbers, IRs, and NNH

In total, 885 women (including 320 with endometriosis) had responded positively to the question ‘Have you been diagnosed with depression requiring treatment’ at least once during the follow-up period. After excluding the misunderstandings from all self-reports and accounting for the first reported event, 308 (1.5%) women with endometriosis out of 21 090, and 535 (0.7%) out of 72 451 without endometriosis reported depression, leading to IRs of 62.2 per 10 000 woman-years (95% CI: 55.4−69.5) and 39.0 per 10 000 woman-years (95% CI: 35.8−42.5), respectively. The ARD was 23.2 per 10 000 woman-years (95% CI: 15.2−30.9). The NNH was 431 (95% CI: 323.7−657.0), meaning that approximately for every 431 women with endometriosis, one additional woman would experience depression requiring treatment compared to those without endometriosis over the same time period.

Among women with endometriosis, 61 (19.1%) of the newly self-reported cases of depressions could be confirmed, while 259 (80.9%) could not be confirmed. For those without endometriosis, 148 (26.2%) of the first self-reported depressions could be confirmed, while 417 (73.8%) could not be confirmed. More details on subcategories and IRs can be found in Table 2.

**Table 2.** Number of events and incidence rates (IRs) of reported depression and other mental health disorders during the 35 months of follow-up, including 95% CIs.

	Endometriosis	No endometriosis
Woman years	49 541	137 137
Number of participants	21 090	72 451
**All self-reported depression**	**320** (100%)	**565** (100%)
IR (95% CI)*	64.6 (57.7–72.1)	41.2 (37.9–44.7)
**All self-reported depression**	**308**	**535**
(excluding misunderstandings)		
IR (95% CI)*	62.2 (55.4–69.5)	39.0 (35.8–42.5)
**Confirmed depression**	**61** (19.1%)	**148** (26.2%)
IR (95% CI)*	12.3 (9.4–15.8)	10.8 (9.1–12.7)
*Thereof*		
Treated by a psychiatrist	52	130
IR (95% CI)*	10.5 (7.8–13.8)	9.5 (7.9–11.3)
Suicide/suicide attempt	2	14
IR (95% CI)*	0.4 (0.0–1.5)	1.0 (0.6–1.7)
Hospital admission	7	4
IR (95% CI)*	1.4 (0.6–2.9)	0.3 (0.1–0.7)
**No confirmed depression**	**259** (80.9%)	**417** (73.8%)
IR (95% CI)*	52.3 (46.1–59.0)	30.4 (27.6–33.5)
*Thereof*		
Treated by a psychologist	26	65
IR (95% CI)*	5.2 (3.4–7.7)	4.7 (3.7–6.0)
Treated by a general practitioner	55	102
IR (95% CI)*	11.1 (8.4–14.5)	7.4 (6.1–9.0)
Treated by neurologist	0	8
IR (95% CI)*	0.0 (0.0–0.0)	0.6 (0.3–1.1)
Potential depression, no medical	57	118
clarification	11.5 (8.7–14.9)	8.6 (7.1–10.3)
IR (95% CI)*		
Depression during pregnancy	0	2
IR (95% CI)*	0.0 (0.0–0.0)	0.1 (0.0–0.5)
Misunderstandings	12	30
IR (95% CI)*	2.4 (1.3–4.2)	2.2 (1.5–3.1)
Other mental health problems ^A^	112	93
IR (95% CI)*	22.6 (18.6–27.2)	6.8 (5.5–8.3)
**All self-reported depression** (in	**62**	**303**
participants without pain, excluding		
misunderstandings)		
IR (95% CI)*	48.4 (37.1–62.1)	32.2 (28.7–36.0)

Only the first reported event per study participant is considered. Bold values are main category values.

* IR and 95% CI are shown per 10 000 woman-years.

A including bipolar or anxiety disorders (including panic attacks), schizophrenia, eating disorders, burnout syndrome, mood changes, depressive mood.

Only 5314 (25.2%) women with endometriosis did not suffer from PP or menstrual pain when starting the OC use, compared to 50 019 (69.0%) women without endometriosis. Of these, women 62 women with endometriosis (IR: 48.4/10 000 woman-years, 95% CI: 37.1−62.1) and 303 women without endometriosis reported depression (IR: 32.2/10 000 woman-years, 95% CI: 28.7−36.0).

### Time-to-event analysis

**Figure 3.**
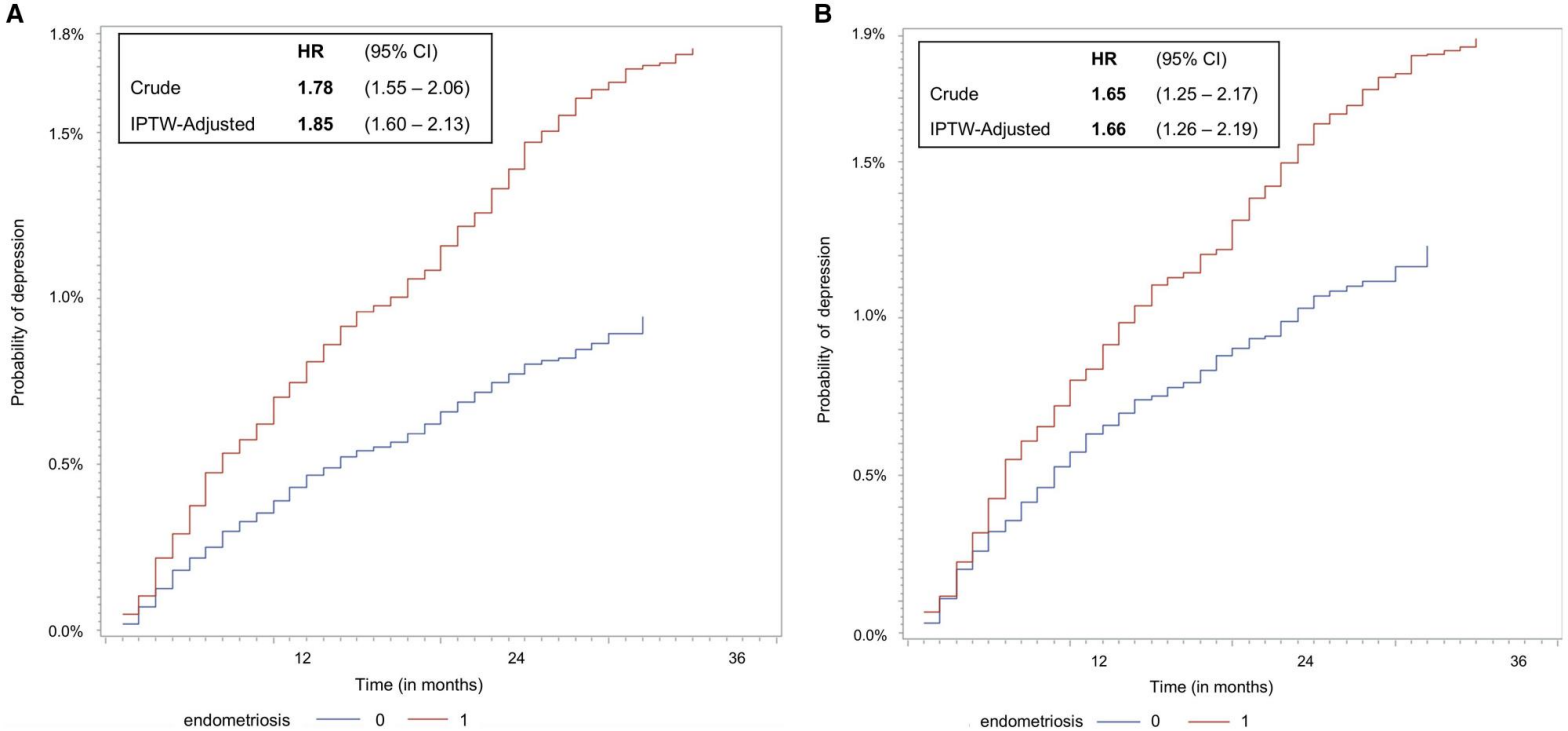
**Time-to-event analysis of the association between endometriosis and depression.** Cumulative incidence of depression in women with and without endometriosis along with crude and inverse probability of treatment (IPT)-weighted hazard ratios (HRs) and CIs in all OC users (**A**) and all OC users without pelvic and menstrual pain (**B**). OC, oral contraceptive.

The mean stabilized weights were 1.0 for both groups and no extreme weight values were observed (Supplementary Table S2), indicating balance between reducing confounding bias and increasing bias and variance (Cole and Hernán, 2008). Figure 3A presents the results of the time-to-event analysis. During the total follow-up of 35 months, OC users with endometriosis had a higher probability of experiencing a depressive event compared to OC users without endometriosis. Overall, women with endometriosis were at a 78% elevated risk of a depressive event compared to those without endometriosis (crude HR 1.78, 95% CI: 1.55–2.06). Observed imbalances in background characteristics between the groups did not explain this excess risk, as the IPT-weighted model including age, BMI, age at menarche, smoking, and education estimated a similar and even slightly greater risk elevation associated with endometriosis (IPT-weighted HR 1.85, 95% CI: 1.60–2.13).

The findings were similar in the subgroup of women being prescribed an OC but not experiencing pelvic or menstrual pain at baseline (Fig. 3B). Overall, women with endometriosis yet no pain had a 65% elevated risk of a depressive event (crude HR 1.65, 95% CI: 1.25–2.19), and when accounting for the same baseline risk factors to obtain a balanced sample with respect to putative confounders, this excess risk remained (IPT-weighted HR 1.66, 95% CI: 1.26–2.19).

### Sensitivity analyses

When restricting the outcome to concern only depression classified as confirmed via medical documentation and independent adjudication by experts in the field, the excess risk to women with endometriosis was smaller (crude HR 1.27, 95% CI: 0.94–1.72), but it did remain substantially elevated when the comparison groups were balanced with respect to baseline risk factors (IPT-weighted HR 1.41, 95% CI: 1.05–1.89). Furthermore, when the exposed group was limited to women with a surgically confirmed endometriosis (n = 2021), their observed risk of developing a depressive event was 3.5-fold times that of women without endometriosis (crude HR 3.47, 95% CI: 2.65–4.54), and 4-fold greater once baseline risk factors were accounted for (IPT-weighted HR 4.13, 95% CI: 3.20–5.34). Lastly, in a subgroup analysis to address potential selection bias, the HRs for new OC users (crude HR 1.85, 95% CI: 1.54–2.24; IPT-weighted HR 1.92, 95% CI: 1.59–2.32) and previous OC users (crude HR 2.00, 95% CI: 1.60–2.51; IPT-weighted HR 2.09, 95% CI: 1.67–2.62) showed consistent results, further supporting the robustness of our findings across different patient profiles (Supplementary Table S3 and Fig. S2).

### Baseline and follow-up factors potentially affecting mental health

We compared baseline characteristics of women with endometriosis who self-reported depression (N = 308) to those without self-reported depression (N = 20 782). Both groups had a similar baseline age and BMI. Women with self-reported depression had a higher proportion of smoking (26.0% vs 19.4%) and were slightly less likely to have higher education levels compared to those without depression. Endometriosis-related symptoms were generally more prevalent in those with depression, including increased rates of pain during intercourse, painful periods, constipation or diarrhea, and tiredness/weakness. Additionally, this group experienced more severe pain, and a greater proportion had undergone surgery for endometriosis at baseline. Treatment patterns showed higher use of non-prescription painkillers and other measures among those with depression (cf Supplementary Table S4).

Available follow-up information on healthcare utilization and newly diagnosed medical conditions showed that across the complete follow-up period, 18.6% of women with endometriosis required hospitalization compared to 7.4% without endometriosis; among these 9.8% and 3.3% of hospitalizations were planned respectively (Supplementary Table S5). Similarly, 11.8% of women with endometriosis underwent any type of surgery compared to 4.1% in the no endometriosis group. Also, reports of regular medication use (other than OC) and newly diagnosed disease were more common in women with endometriosis (26.3% and 18.5% respectively compared to 16.1% and 8.7% in women without endometriosis).

## Discussion

In our large longitudinal population-based study of women using OC, we found that endometriosis increased the risk of developing a depressive event, although the observed absolute risk increase in both groups was small. After adjusting for observed differences in baseline risk factors such as age, BMI, age at menarche, smoking, and education, the risk of a self-reported depressive event in women with endometriosis was increased by 85% compared to women without endometriosis. This finding was supported by sensitivity analyses restricted to (i) confirmed depression to account for potential outcome reporting bias, (ii) women with surgically confirmed endometriosis to minimize exposure misclassification bias, and (iii) subgroups of women with and without prior use of OC to account for potential selection bias.

Previous studies have found higher levels of depression in women with endometriosis compared with women without endometriosis, with most of the observed effect mediated by pelvic pain (Chen *et al.*, 2016; Laganà *et al.*, 2017; Gambadauro *et al.*, 2019). By conditioning on pain symptoms in both women with endometriosis and those without, the observed associations were noticeably smaller to absent (Gambadauro *et al.*, 2019). However, we found evidence that endometriosis still has a significant effect on mental health on women also under treatment with OC. In contrast to prior studies, our findings were similar also in women that were free of pelvic or menstrual pain at baseline, indicating that factors other than pain might contribute to poor mental health in women living with endometriosis (e.g. the occurrence of comorbidities which could impact quality of life). Although our study did not directly measure pain levels throughout the follow-up period, the increased rates of medical interventions and regular medication use among women with endometriosis could reflect ongoing disease activity and persistent symptoms, including pain, which could exacerbate mental health challenges, and require more research. As reported in the literature, a combination of multiple factors considerably impacts the patient’s emotional and physical well-being (Fourquet *et al.*, 2010). This includes for example the often complex and individual treatment regime (depending on patient profile, disease characteristics, side effects, pregnancy wish) which does not always lead to successful symptom relief, other symptoms affecting mental health (e.g. infertility), the patient’s awareness of endometriosis as a chronic disease and its possible disease progression, an elevated risk of other diseases, lack of support in the patient’s community, and the symptom-normalization by healthcare practitioners. A recent systematic review (Zippl *et al.*, 2024) underscores the role of chronic pelvic pain as a central mediator in the development of depression among individuals with endometriosis. However, the study also highlights other influential factors, such as a shared genetic predisposition between endometriosis and mental health disorders, which may independently contribute to the increased risk of depression and anxiety in this population. Furthermore, studies have shown that OC use may increase the risk of depression, especially during the first 2 years of use or when initiated during adolescence (Johansson *et al.*, 2023). Since OCs are listed as a guideline recommended medical treatment for relieving endometriosis-associated pain, it is crucial for healthcare providers to inform patients with endometriosis about the potential for a slight increase in mood-related side effects when using OCs. Close monitoring by healthcare providers is essential, and treatment plans should be adjusted if necessary to ensure patient well-being. By contextualizing the small absolute increase in depression risk, as seen in our study, physicians can help mitigate concerns and avoid unnecessary undertreatment of endometriosis, which could lead to worse outcomes such as increased surgical morbidity or use of more expensive and potentially less safe medications. To support clinical decision-making, a patient decision aid was added in the Supplementary Data File S1 and can be used in practice. Overall, living with endometriosis is a complex individual multifactorial disease experience, and further research is required (i) to explore effective treatment strategies to improve mental health beyond antidepressive medication, (ii) to understand the full spectrum of factors that could impact overall well-being, and (iii) to assess the impact of other (alternative) interventions to alleviate endometriosis-related problems that negatively affect mental health.

### Strengths and limitations

This study has several strengths. First, we could include a large number of participants to obtain good statistical power. The large sample size also allowed analysis of different subgroups to increase compatibility with other research on endometriosis and depression. Secondly, this study aimed to accurately represent and obtain an understanding of the patient perspective of mental health by primarily focusing on all self-reported depression. Thirdly, we could incorporate data from diverse geographical regions across and within the included study countries, leading to better generalizability of the findings. Additionally, to estimate the causal effect of endometriosis on depression, we used modern epidemiological methods (i.e. IPTW) to account for measured confounding. Lastly, the researchers used a causal framework for identification of confounding variables. Variables that were considered as colliders or mediators were not included in the statistical model to reduce the risk of bias.

This study also has some limitations which should be considered when interpreting the research findings. First, the results of this study can only be generalized to women being treated with OCs. Data on the exposure to other endometriosis treatments were not considered and therefore caution should be exercised when extrapolating these findings to women exposed to other treatments. Secondly, while we used IPTW to address confounding, this requires measures of the putative confounders and the potential for unmeasured confounding thus remains. Despite employing rigorous methodologies, the risk of model misspecification persists, which could lead to biased estimates and affect the accuracy of our conclusions. Additionally, it is possible that other factors not included in our analysis might have influenced the observed association between endometriosis and depression. Genetic predispositions are known to contribute significantly to both conditions. Although our study did not directly explore specific genes or pathways, existing literature suggests the involvement of genes related to common genetic pathways in both endometriosis and depression (Adewuyi *et al.*, 2021; Koller *et al.*, 2023). The potential shared genetic influences between these traits and other unmeasured or still unknown shared causes might have influenced our results, and the extent of their impact remains an area for future research. Thirdly, we recognize the presence of potential differences in the prevalence and diagnosis of depression across the different study countries. While a sensitivity analysis was conducted to consider only depression classified as confirmed, variation in depression reporting by participants from different countries may still exist. Lastly, potential misclassification of endometriosis could introduce bias. Failing to classify women with undiagnosed or asymptomatic endometriosis as exposed could lead to an underestimation of the true association, which would depend on the likelihood of depression among these misclassified women. Overall, due to the potential for unmeasured confounding and residual bias, we are unable to draw causal conclusions from the observed associations, despite their statistical significance.

## Conclusions

Among women receiving OCs, endometriosis has an adverse influence on mental health. The absolute risk increase remains small, and the tradeoff between managing endometriosis symptoms and potential mood-related side effects should be carefully considered by both patients and healthcare providers. Our findings suggest that factors other than pelvic and menstrual pain may contribute to the deterioration of mental health in individuals with endometriosis when using OCs. To fully comprehend the array of factors contributing to mental health issues in individuals living with endometriosis, further research is necessary. There also should be further strong efforts to find tailored interventions to improve mental health and overall quality of life outcomes among women with endometriosis.

## Supplementary Material

deae299_Supplementary_Data_File_S1

deae299_Supplementary_Figure_S1

deae299_Supplementary_Figure_S2

deae299_Supplementary_Table_S1

deae299_Supplementary_Table_S2

deae299_Supplementary_Table_S3

deae299_Supplementary_Table_S4

deae299_Supplementary_Table_S5

## Data Availability

The data underlying this article were provided by ZEG Berlin, Bayer Pharmaceuticals, Merck Sharp & Dohme LLC, and Theramex by permission. Data will be shared on request to the corresponding author with permission of the listed owners of the data.
