# Correction to: Endometriosis and risk of depression among oral contraceptive users: a pooled analysis of cohort studies from 13 countries

**DOI:** 10.1093/humrep/deaf065

**Published:** 2025-04-03

This is a correction to: P De Corte, I Milhoranca, A S Oberg, T Kurth, S Mechsner, K Heinemann, Endometriosis and risk of depression among oral contraceptive users: a pooled analysis of cohort studies from 13 countries, *Human Reproduction*, Volume 40, Issue 3, March 2025, Pages 479–486, https://doi.org/10.1093/humrep/deae299

In the originally published version of this manuscript, one of corresponding author, Pauline De Corte, affiliations was erroneously omitted. This article has been updated with both affiliations.

1. Berlin Center for Epidemiology and Health Research, Berlin, Germany

And

2. Institute of Public Health, Charité – Universitätsmedizin Berlin, Berlin Germany

