# Supplementary material for: Endometriosis and risk of depression among oral contraceptive users: a pooled analysis of cohort studies from 13 countries
Source: Hum Reprod. 2025 Jan 12;40(3):479–86. doi: 10.1093/humrep/deae299 (PMC11879161; doi:10.1093/humrep/deae299)

Supplementary Data File S1: Patient Decision Aid

WHAT IS THE RISK OF NEW-ONSET DEPRESSION WHEN USING THE COMBINED ORAL CONTRACEPTIVES TO TREAT SYMPTOMS ASSOCIATED WITH ENDOMETRIOSIS?

Combined oral contraceptive pills ("the pill") are often used to relieve pain symptoms associated with endometriosis. While combined pills can be effective in about two-thirds of the cases, on the other hand, adverse events can occur, although the pill is generally considered safe when used by women without major contraindications.

Depression is one of the side effects reported by some women using the pill. This may be important because women with endometriosis may be more likely to develop depression than women without endometriosis, mainly because of chronic pain and worsened quality of life.

Therefore, some information can help you understand the additional risk of developing depression if you decide to use a combined oral contraceptive to reduce your symptoms caused by endometriosis.

Based on the findings of reliable studies, among women who have never experienced depression previously, the likelihood of developing depression while using the combined pill is about 0.7% for women without endometriosis, and it is 1.5% for those who also have endometriosis. This means that having endometriosis confers an additional 0.8% chance of developing depression while using the pill. To put it the other way round, if you have endometriosis, you have a 98.5% chance of NOT developing depression while on the pill, rather than a 99.3% chance.

In fact, compared to women without endometriosis, for every 431 women with endometriosis who start using the pill, about one additional woman would develop depression requiring treatment. Depression caused by the pill usually resolves within a few months of stopping its use.

The figure below shows the actual number of new cases of depression per 10,000 women with or without endometriosis taking the combined pill for one year. In summary, for every 10,000 women with endometriosis who use the pill, 23 more will develop depression compared with women without endometriosis. For simplicity, the risk refers to women aged 30 in both groups, although the available data are from groups with slightly different average ages.

This information can help you and your healthcare professional decide what is best for you.

Risk of depression by age 30 in combined pill users withouth endometriosis:

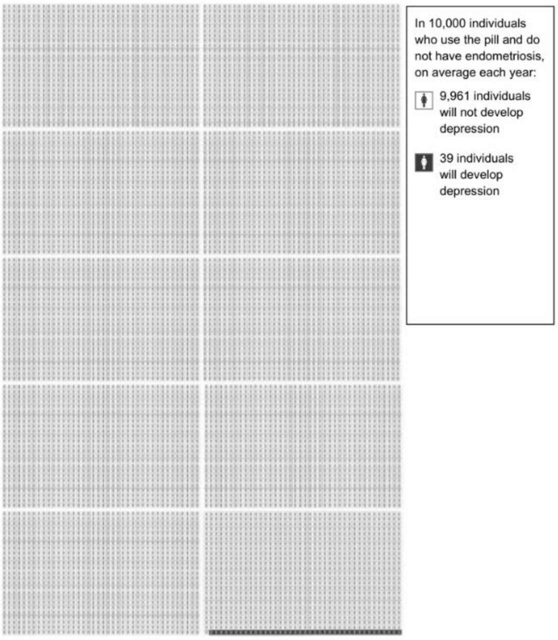

Risk of depression by age 30 in combined pill users with endometriosis:

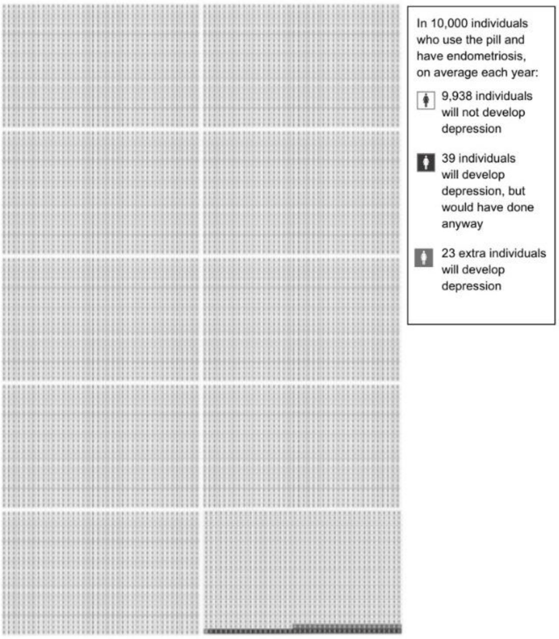

Supplement: deae299_Supplementary_Data_File_S1 [file deae299_supplementary_data_file_s1.pdf]
