# Supplementary material for: Endometriosis and risk of depression among oral contraceptive users: a pooled analysis of cohort studies from 13 countries
Source: Hum Reprod. 2025 Jan 12;40(3):479–86. doi: 10.1093/humrep/deae299 (PMC11879161; doi:10.1093/humrep/deae299)
Supplement: deae299_Supplementary_Figure_S1 [file deae299_supplementary_figure_s1.pdf]

OXFORD
human reproduction
Human Reproduction, 2025, 1–1
https://doi.org/10.1093/humrep/deae299
Supplementary Data

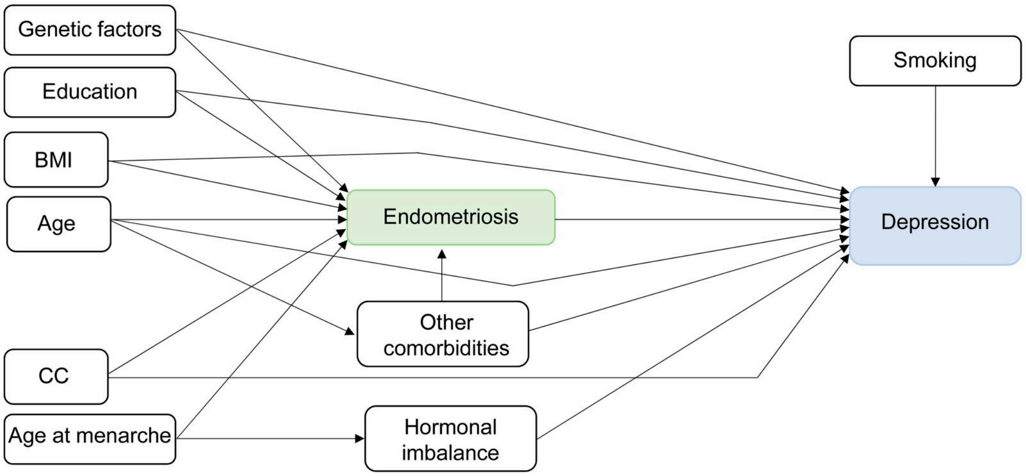

Used abbreviations: BMI = Body Mass Index, CC = Common Causes

Supplementary Figure S1. Causal diagram describing the hypothesized relationship between endometriosis and depression, with all of their common causes (CCs) and a putative mediator.
