# Supplementary material for: Endometriosis and risk of depression among oral contraceptive users: a pooled analysis of cohort studies from 13 countries
Source: Hum Reprod. 2025 Jan 12;40(3):479–86. doi: 10.1093/humrep/deae299 (PMC11879161; doi:10.1093/humrep/deae299)
Supplement: deae299_Supplementary_Figure_S2 [file deae299_supplementary_figure_s2.pdf]

Human Reproduction, 2025, 1–1
https://doi.org/10.1093/humrep/deae299
Supplementary Data

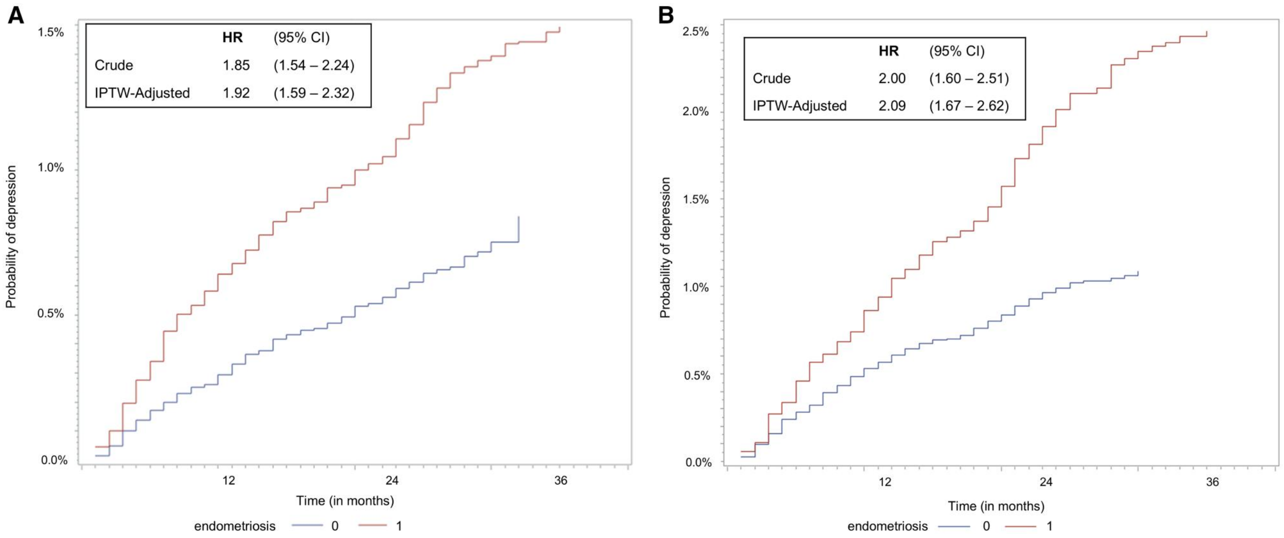

Supplementary Figure S2. Time-to-event analysis of the association between endometriosis and depression. Cumulative incidence of depression in women with and without endometriosis along with crude and IPT-weighted HRs and CIs in first-time OC users (S2.1) and previous OC users (S2.2). HR, hazard ratio; IPTW, inverse probability of treatment weighting; OC, oral contraceptive.
