# Supplementary material for: Endometriosis and risk of depression among oral contraceptive users: a pooled analysis of cohort studies from 13 countries
Source: Hum Reprod. 2025 Jan 12;40(3):479–86. doi: 10.1093/humrep/deae299 (PMC11879161; doi:10.1093/humrep/deae299)
Supplement: deae299_Supplementary_Table_S1 [file deae299_supplementary_table_s1.pdf]

Human Reproduction, 2025, 1–1
https://doi.org/10.1093/humrep/deae299
Supplementary Data

Supplementary Table S1. Overview of the data sources used for the pooled analysis.

Study	INAS-VIPOS	PRO-E2
**Population**	Women with endometriosis only with a newly prescribed hormonal treatment	All women prescribed with a newly prescribed hormonal treatment
**Study period**	2010–2017	2014–2019
**Countries**	Germany, Poland, Russia, Hungary, Switzerland, and Ukraine	Australia, Austria, Colombia, France, Germany, Hungary, Italy, Poland, Russia, Spain, and Sweden
**Total study population**	27 840	101 498
**Follow-up timepoints**	6, 12, 24, 48, 60, 72, 84 months after study entry	6, 12, and 24 months after study entry
