# Supplementary material for: Endometriosis and risk of depression among oral contraceptive users: a pooled analysis of cohort studies from 13 countries
Source: Hum Reprod. 2025 Jan 12;40(3):479–86. doi: 10.1093/humrep/deae299 (PMC11879161; doi:10.1093/humrep/deae299)
Supplement: deae299_Supplementary_Table_S2 [file deae299_supplementary_table_s2.pdf]

Human Reproduction, 2025, 1–1
https://doi.org/10.1093/humrep/deae299
Supplementary Data

Supplementary Table S2. Mean, minimum, and maximum of the inverse probability of treatment weights.

	All OC users	
	**Endometriosis**	**No endometriosis**
Estimated weights		
Mean	1.00	1.00
Minimum	0.42	0.85
Maximum	2.27	1.66

OC, oral contraceptive.
