# Supplementary material for: Endometriosis and risk of depression among oral contraceptive users: a pooled analysis of cohort studies from 13 countries
Source: Hum Reprod. 2025 Jan 12;40(3):479–86. doi: 10.1093/humrep/deae299 (PMC11879161; doi:10.1093/humrep/deae299)
Supplement: deae299_Supplementary_Table_S3 [file deae299_supplementary_table_s3.pdf]

OXFORD
human reproduction

*Human Reproduction*, 2025, 1–1
https://doi.org/10.1093/humrep/deae299
Supplementary Data

**Supplementary Table S3.** Sensitivity analyses for the relationship between endometriosis and depression; crude and IPTW-adjusted HRs with 95% CIs.

| | HR | (95% CI) |
|---|---|---|
| **Sensitivity analysis I: expert-review confirmed depression (N = 209)** | | |
| Crude | 1.27 | (0.94–1.72) |
| IPTW-adjusted | 1.41 | (1.05–1.89) |
| **Sensitivity analysis II: surgically confirmed endometriosis (N = 2021)** | | |
| Crude | 3.47 | (2.65–4.54) |
| IPTW-adjusted | 4.13 | (3.20–5.34) |

IPTW, inverse probability of treatment weighting; HR, hazard ratio; N, number.
