# Supplementary material for: Endometriosis and risk of depression among oral contraceptive users: a pooled analysis of cohort studies from 13 countries
Source: Hum Reprod. 2025 Jan 12;40(3):479–86. doi: 10.1093/humrep/deae299 (PMC11879161; doi:10.1093/humrep/deae299)
Supplement: deae299_Supplementary_Table_S4 [file deae299_supplementary_table_s4.pdf]

OXFORD
human reproduction

Human Reproduction, 2025, 1–1
https://doi.org/10.1093/humrep/deae299
Supplementary Data

Supplementary Table S4. Baseline characteristics of endometriosis patients with and without depression.

	Endometriosis		Endometriosis	
	No self-reported depression		Self-reported depression	
**Number of participants**	N = 20 782		N = 308	
**Demographics and lifestyle**				
Age at baseline	32.8	±8.91	32.4	±9.57
BMI at baseline	23.4	±4.24	23.4	±4.37
Smoking	4038	(19.4%)	80	(26.0%)
Education: higher than university level	9551	(46.0%)	127	(41.2%)
**Gynecological history**				
Age at menarche	12.9	±1.32	12.8	±1.30
Gravidity	11 630	(56.0%)	169	(54.9%)
Parity	10 739	(51.7%)	154	(50.0%)
**Endometriosis history**				
Timespan first symptoms—first diagnosis (months)	8.8	±21.6	14.7	±26.22
Endometriosis of first grade relatives	3375	(16.2%)	58	(18.8%)
Operation to diagnose or treat endometriosis	2243	(10.8%)	68	(22.1%)
Of which (multiple nominations possible):				
Diagnostic laparoscopy or laparotomy	767	(3.7%)	24	(7.8%)
Therapeutic laparoscopy	1152	(5.5%)	40	(13.0%)
Excision of lesions/adhesions	204	(1.0%)	15	(4.9%)
Removal of ovarian cysts	320	(1.5%)	10	(3.3%)
Removal of ovary/fallopian tubes	69	(0.3%)	3	(1.0%)
Hysterectomy	11	(0.1%)	0	(0.0%)
**Endometriosis reported symptoms**				
Pelvic pain	6345	(30.5%)	114	(37.0%)
Pain during/after intercourse	4319	(20.8%)	88	(28.6%)
Difficulty conceiving/infertility	2420	(11.6%)	37	(12.0%)
Painful periods	11 621	(55.9%)	192	(62.3%)
Heavy or irregular bleeding	9804	(47.2%)	152	(49.4%)
Pain when passing urine	1584	(7.6%)	26	(8.4%)
Pain when opening bowels	1461	(7.0%)	34	(11.0%)
Constipation or diarrhea	2086	(10.0%)	57	(18.5%)
Tiredness/weakness	4983	(24.0%)	120	(39.0%)
Other endometriosis-related symptoms	462	(2.2%)	11	(3.6%)
**Endometriosis-related pain**				
At least one selected pain symptom	15 398	(74.1%)	247	(80.2%)
All three pain symptoms selected	1193	(5.7%)	41	(13.3%)
Pain preventing from work	5213	(25.1%)	100	(32.5%)
Pain scale category 1 (mild)	8148	(39.2%)	106	(34.4%)
Pain scale category 2 (moderate)	8625	(41.5%)	132	(42.9%)
Pain scale category 3 (severe)	1673	(8.1%)	43	(14.0%)
**Medical history**				
Any surgery (other than endometriosis-related)	3886	(18.7%)	78	(25.3%)
Take medication on regular basis	2372	(11.4%)	53	(17.2%)
Any other serious disease	833	(4.0%)	14	(4.6%)
**Current endometriosis treatment (excl. OCs)**				
Other measures to treat endometriosis	10 696	(51.5%)	196	(63.6%)
Of which (multiple nominations possible):				
Non-prescription painkillers	7940	(38.2%)	145	(47.1%)
Herbal products/acupuncture	2598	(12.5%)	50	(16.2%)
Dietary modifications	1761	(8.5%)	38	(12.3%)
Home remedies/manual therapy	3604	(17.3%)	80	(26.0%)
**Treatment history (before current OC)** (multiple nominations possible):				
Ever use of oral contraceptives	5038	(24.2%)	107	(34.7%)
Combined hormonal contraceptives	2571	(12.4%)	48	(15.5%)
Progestogen only preparations	699	(3.4%)	22	(7.1%)
GnRH analog	231	(1.1%)	6	(2.0%)
Danazol	62	(0.3%)	3	(1.0%)
Painkillers	741	(3.6%)	16	(5.2%)
Phytohormones/herbal medication	91	(0.4%)	1	(0.3%)
Other, unspecified	51	(0.3%)	3	(1.0%)

OC, oral contraceptive; N, number.
