# Supplementary material for: Endometriosis and risk of depression among oral contraceptive users: a pooled analysis of cohort studies from 13 countries
Source: Hum Reprod. 2025 Jan 12;40(3):479–86. doi: 10.1093/humrep/deae299 (PMC11879161; doi:10.1093/humrep/deae299)
Supplement: deae299_Supplementary_Table_S5 [file deae299_supplementary_table_s5.pdf]

Human Reproduction, 2025, 1–1
https://doi.org/10.1093/humrep/deae299
Supplementary Data

Supplementary Table S5. Follow-up events that could contribute to the risk of developing depression.

	Endometriosis		No Endometriosis	
	N = 21 090		N = 72 451	
Hospitalization	3920	(18.6%)	5326	(7.4%)
Planned	2072	(9.8%)	2423	(3.3%)
Including any type of surgery	2493	(11.8%)	2952	(4.1%)
Surgery because of endometriosis	1622	(7.7%)	NA	–
Regular use of medication (excl. OC use)	5542	(26.3%)	11 696	(16.1%)
Any new self-reported disease	3909	(18.5%)	6300	(8.7%)

OC, oral contraceptive; N, number.

© The Author(s) 2025. Published by Oxford University Press on behalf of European Society of Human Reproduction and Embryology.
This is an Open Access article distributed under the terms of the Creative Commons Attribution License (https://creativecommons.org/licenses/by/4.0/), which permits unrestricted reuse, distribution, and reproduction in any medium, provided the original work is properly cited.
Human Reproduction, 2025, 00, 1–15
https://doi.org/10.1093/humrep/deae299
Original Article
